# Extremely short duration high intensity interval training substantially improves insulin action in young healthy males

**DOI:** 10.1186/1472-6823-9-3

**Published:** 2009-01-28

**Authors:** John A Babraj, Niels BJ Vollaard, Cameron Keast, Fergus M Guppy, Greg Cottrell, James A Timmons

**Affiliations:** 1 Translational Biomedicine, School of Engineering and Physical Sciences, Heriot-Watt University Edinburgh, Scotland, UK; 2 The Wenner-Gren Institute, Arrhenius Laboratories, Stockholm University, Sweden

## Abstract

**Background:**

Traditional high volume aerobic exercise training reduces cardiovascular and metabolic disease risk but involves a substantial time commitment. Extremely low volume high-intensity interval training (HIT) has recently been demonstrated to produce improvements to aerobic function, but it is unknown whether HIT has the capacity to improve insulin action and hence glycemic control.

**Methods:**

Sixteen young men (age: 21 ± 2 y; BMI: 23.7 ± 3.1 kg·m^-2^; VO_2_peak: 48 ± 9 ml·kg^-1^·min^-1^) performed 2 weeks of supervised HIT comprising of a total of 15 min of exercise (6 sessions; 4–6 × 30-s cycle sprints per session). Aerobic performance (250-kJ self-paced cycling time trial), and glucose, insulin and NEFA responses to a 75-g oral glucose load (oral glucose tolerance test; OGTT) were determined before and after training.

**Results:**

Following 2 weeks of HIT, the area under the plasma glucose, insulin and NEFA concentration-time curves were all reduced (12%, 37%, 26% respectively, all P < 0.001). Fasting plasma insulin and glucose concentrations remained unchanged, but there was a tendency for reduced fasting plasma NEFA concentrations post-training (pre: 350 ± 36 v post: 290 ± 39 μmol·l^-1^, P = 0.058). Insulin sensitivity, as measured by the Cederholm index, was improved by 23% (P < 0.01), while aerobic cycling performance improved by ~6% (P < 0.01).

**Conclusion:**

The efficacy of a high intensity exercise protocol, involving only ~250 kcal of work each week, to substantially improve insulin action in young sedentary subjects is remarkable. This novel time-efficient training paradigm can be used as a strategy to reduce metabolic risk factors in young and middle aged sedentary populations who otherwise would not adhere to time consuming traditional aerobic exercise regimes.

## Background

The prevalence of type 2 diabetes (T2D) is rapidly increasing world-wide and, for example, in the United States it reached 17.5 million people in 2007 [1]. Aside from the associated reduction in quality-of-life and the increase in morbidity and mortality for the affected individuals, the economic burden was estimated at $116 billion in excess medical expenditures and $58 billion in reduced productivity [1]. Similarly, the estimated direct and indirect economic costs of cardiovascular disease (CVD) in the US for 2008 are estimated at $287 billion [2].

The risk of developing CVD and T2D can be modified by regular physical activity [3]. However, there is no consensus on the nature of exercise therapy required to provide adequate health benefits, particularly with regard to the volume-intensity relationship. Furthermore, as we do not understand the precise mechanisms which link physical activity and a reduced risk of developing CVD or T2D, the scientific rationale for current health guides needs to be improved [4]. For exercise guidelines to yield a positive economic benefit for society, as well as a health benefit for the individual, not only should the regime reliably modify key disease risk factors, it must also be plausible to implement.

Metabolic adaptations associated with traditional aerobic exercise training correlate with improved insulin action and glycemic control [5,6]. These effects appear to be independent of changes in body composition [7], and some evidence suggests that greater improvements in insulin sensitivity may be achieved with higher training intensities [8-10]. Current recommendations for improving glycemic control involve performing moderate to vigorous intensity aerobic and resistance exercise for several hours per week [11,12]. However, the general population fails to follow such regimes due to lack of time, motivation and adherence [13]. This suggests that the current focus on time-consuming moderate intensity physical activity, aimed at increasing total energy expenditure, may not be optimal for reducing the risk of developing T2D.

Recently an extremely low volume high-intensity interval training paradigm (HIT), consisting of no more than 7.5 minutes of exercise per week, has been proposed as a novel, time-efficient exercise regime for improving aerobic fitness [4,14]. We speculated that it should be possible to substantially improve insulin action using HIT despite a negligible contribution to total energy expenditure as this training model would substantially reduce muscle glycogen stores. Compared to traditional strategies for reduction of risk factors of CVD and T2D, the extremely low volume of exercise required with HIT may promote adherence and thus represent a genuinely preventative public health strategy.

## Methods

### Subjects

Twenty-five young healthy sedentary or recreationally active men were recruited to participate in this study, with none engaged in a structured endurance training program. Subjects were randomly allocated to one of two parts of the study. Sixteen subjects (mean ± SD: 21 ± 2 y; 82 ± 17 kg; 1.83 ± 0.08 m; BMI: 23.7 ± 3.1 kg·m^-2^; VO_2_max: 48 ± 9 ml·kg^-1^·min^-1^) were allocated to the main part of the study, and completed the full experimental procedures. The remaining nine subjects (mean ± SD: 23 ± 8 y; 73 ± 9 kg; 1.78 ± 0.09 m; 23.0 ± 1.4 kg·m^-2^; VO_2_max: 47 ± 11 ml·kg^-1^·min^-1^) took part in a separate experiment to determine intra-individual variation in response to an oral glucose tolerance test, and did not perform HIT. There were no significant differences in the age, height, weight, BMI or VO_2_max between the two groups of subjects. Subjects were informed of the experimental protocol both verbally and in writing before giving informed consent. Furthermore, all subjects were informed about how potential life-style changes could affect the results of the study, and were requested to maintain their normal diet and levels of physical activity (apart from the training program) throughout the duration of the study. The study protocol was approved by the institutional Ethics Committee and conducted in accordance with the Helsinki Declaration.

### Experimental procedures

Baseline aerobic performance and health parameters were determined over a 2-week period prior to commencement of the training program.

### Oral glucose tolerance test (OGTT)

Subjects refrained from performing any strenuous physical activity for 2 days prior to the OGTT, and attended the laboratory having fasted overnight. Venous blood samples were collected by venepuncture before, and 60, 90 and 120 min after ingestion of 75 g glucose (Fisher Scientific, Loughborough, UK) dissolved in 100 ml of water. Plasma was separated by centrifugation (10 min at 1600 g) and stored at -20°C until analysis of glucose, insulin and NEFA concentrations. Plasma glucose concentrations were measured using an automatic analyzer (YSI Stat2300, Yellow Spring Instruments, Yellow Spring, OH) and plasma insulin concentrations were determined by ELISA (Invitrogen, UK). Plasma insulin was measured only for samples taken at 0, 60, and 90 min. Plasma NEFA concentrations were determined by a colorimetric assay (Wako Chemicals, Germany) using a modified protocol. Briefly, 3.75 μl of plasma samples and standards of known concentration were pipetted into a 96-well plate. 75 μl of colour reagent A was added to each well and incubated at 37°C for 10 min. 150 μl of colour reagent B was added and incubated for a further 10 min at 37°C. The plate was then removed from the incubator and allowed to cool to room temperature prior to the absorbance being read at 550 nm. Coefficients of variation (CV) for duplicate samples were 3% for glucose, 5% for insulin, and 8% for NEFAs.

### VO_2_peak test

On a separate occasion, subjects performed an exhaustive incremental cycling test (Lode Excalibur Sport, Groningen, the Netherlands) to determine maximal power output (Wmax) and maximal oxygen uptake capacity (VO_2_peak) using an online gas analysis system (SensorMedics, Bilthoven, the Netherlands). After cycling at 30 W for 1 min, power output was increased by 30 W·min^-1 ^until volitional exhaustion. VO_2_peak was determined as the highest value achieved over a 20-s period.

### Time trials

Endurance performance was determined to provide an integrated physiological readout to facilitate comparison of the present study with previous studies which provided data from muscle biopsy samples [15]. Subjects performed two self-paced cycling time trials in which they were instructed to complete 250 kJ of work as fast as possible. The linear factor was chosen to produce a power output corresponding to 75% of Wmax at a pedal rate of 90 rpm. No encouragement was given, and subjects were blinded from information on time, power output and pedal frequency. The amount of work (kJ) completed was called out every 25 kJ. Time trials were spaced at least two days apart. Although the first of the two pre-training trials was mainly used as a familiarisation trial, the fastest time achieved in the two trials was considered to best represent the pre-training performance level and used in the analysis (19 out of 25 subjects performed better in the second trial than in the familiarisation trial).

### Sprint interval training

The sprint training protocol was similar to that used previously by Burgomaster *et al*. [4,14]. Six sessions of sprint interval exercise were spread over 14 days, with 1 or 2 days of rest between each session. Each training session consisted of 4–6 repeated 30-s all-out cycling efforts against a resistance equivalent to 7.5% of body weight (Wingate tests), with 4 min of recovery between sprints. During recovery, subjects remained on the bike and either rested or cycled at a low cadence without resistance. The number of sprints increased from 4 during the first two sessions, to 5 in the third and fourth sessions, and 6 in the last two sessions. Total time commitment was 17–26 min per session, involving only 2–3 minutes sprint exercise.

### Post-training assessment

A second OGTT was performed after completion of the training program. In order to determine whether potential changes were due to acute effects attributable to the last training session, subjects were tested either two (n = 10), or three (n = 6) days after the last bout of exercise. One day after the second OGTT subjects performed a third cycling time trial in order to determine changes in aerobic performance.

### Intra-individual variability in time trial performance and OGTT-response

In order to assess normal intra-individual variation in response to an OGTT over a period of several weeks as used in the present study, nine subjects performed an identical protocol to the training group but without performing the six training sessions. Coefficients of variation (CV) for repeated measurements within individual subjects were determined for baseline glucose and NEFA levels, for glucose and NEFA area under the plasma curve, and for time trial performance.

### Calculations and statistical analysis

Area under the plasma curve (AUC) was calculated using the conventional trapezoid rule. The Cederholm index, which represents peripheral insulin sensitivity [16], was calculated using the formula:

ISI_Cederholm _= 75000 + (G_0_-G_120_) × 1.15 × 180 × 0.19 × BW/120 × G_mean _× log (I_mean_)

Where BW is body weight, G_0 _and G_120 _are plasma glucose concentration at 0 and 120 min (mmol·l^-1^), and I_mean _and G_mean _are the mean insulin (mU·l^-1^) and glucose (mmol·l^-1^) concentrations during the OGTT.

All data are presented as means ± SEM. Plasma glucose, insulin, and NEFA responses to the pre-training and post-training OGTTs were analyzed using two-way repeated measures ANOVA with *post hoc *Student Newman-Keuls tests. Differences between pre-training and post-training data for time trial performance, AUCs for plasma glucose, insulin, and NEFA levels, and insulin sensitivity as measured by the Cederholm index were analyzed using paired sample t-tests. Pearson's correlation coefficient was used to assess bivariate correlations between baseline values of, and changes in the variables performance, AUCs of glucose, insulin, and NEFAs, and the Cederholm Index. Significance was accepted at P < 0.05.

## Results

### Glucose responses

**Figure 1:**
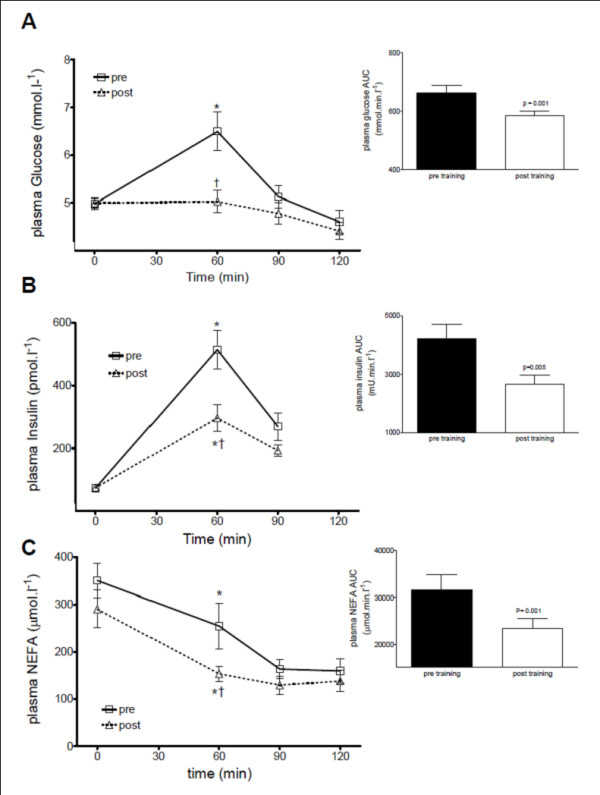
**Response to an oral glucose load pre- and post-HIT**. Plasma concentrations and AUC for A: glucose, B: insulin and C: NEFAs. (*: P < 0.01 for 0 min v 60 min; †: P < 0.01 for pre v post).

Fasting plasma glucose concentrations were unaltered after 2 weeks of HIT. In the pre-training OGTT, plasma glucose concentration was elevated 60 min after the 75 g glucose bolus (Figure 1A; 0 min: 5.0 ± 0.1 v 60 min: 6.5 ± 0.4 mmol·l^-1^; P < 0.0001), but not post-HIT (Figure 1A; 0 min: 5.0 ± 0.1 v 60 min: 5.0 ± 0.2 mmol·l^-1^). The plasma glucose area under the curve (AUC) was significantly reduced post-training (Figure 1A: AUC pre 664 ± 103 v AUC post 585 ± 65 mmol·min·l^-1^; P < 0.001).

### Insulin responses

Fasting plasma insulin concentrations were unaltered after 2 weeks of HIT. In the pre-training OGTT, plasma insulin concentration was elevated 60 min after the 75-g glucose bolus (Figure 1B; 0 min: 10.5 ± 1.6 v 60 min: 74.0 ± 8.9 mU·l^-1^; P < 0.05). Post-training this increase was significantly attenuated (Figure 1B; 0 min: 10.6 ± 1.6 v 60 min: 42.6 ± 6.0 mU·l^-1^; P < 0.01). Although plasma insulin levels were still elevated 90 min after the 75-g glucose bolus both pre- and post-training, levels were no longer significantly higher than at 0 min (pre: 38.8 ± 6.3 mU·l^-1^; post: 27.7 ± 2.7 mU·l^-1^). The plasma insulin AUC was significantly reduced post-training (Figure 1B: AUC pre 4226 ± 1912 v AUC post 2654 ± 1252 mU·min·l^-1^; P < 0.001). Insulin sensitivity significantly improved following 2 weeks of HIT (Cederholm index: pre 80 ± 6 v post 98 ± 5 mg·l^2^·mmol^-1^·mU^-1^·min^-1^, P < 0.01).

### NEFA responses

There was a trend towards a decrease in baseline plasma NEFA concentrations following HIT (Figure 1C; pre: 350 ± 36 μmol·l^-1 ^v post: 290 ± 39 μmol·l^-1^, P = 0.058). In the pre-training OGTT, plasma NEFA concentration was decreased 60 min after the 75 g glucose bolus (Figure 1C; 0 min: 350 ± 36 μmol·l^-1 ^v 60 min: 255 ± 48 μmol·l^-1^; P < 0.01), and to a greater extent post-HIT (Figure 1C; 0 min: 290 ± 39 μmol·l^-1 ^v 60 min: 153 ± 17 μmol·l^-1^, P < 0.001; pre 60 min: 255 ± 48 μmol·l^-1 ^v post 60 min: 153 ± 17 μmol·l^-1 ^P < 0.05). The plasma NEFA AUC was significantly reduced post-training (Figure 1C: AUC pre 31584 ± 13205 v AUC post 23370 ± 8630 μmol·min·l^-1^; P < 0.001), whereas despite the decreased insulin AUC post-training the incremental NEFA AUC was similar pre- and post-training (pre: -12748 ± 2752 μmol·min·l^-1^; post: -13513 ± 2541 μmol·min·l^-1^).

### Physiological considerations

To bench-mark our results with recently published studies, we determined the impact of HIT on performance. Work done in the 250-kJ time trial was significantly increased by an average of 6% following 2 weeks of HIT (mean difference: 75 s, 95% CI: 21–126 s; P < 0.01). At baseline, the only physiological parameter which related to a metabolic parameter, was aerobic capacity vs. NEFA response to the OGTT (R^2 ^= 0.43, P < 0.001). There was a modest correlation between changes in glucose and insulin AUC (R^2 ^= 0.25, P < 0.05). Changes in performance did not correlate with baseline values for, or changes in the AUCs of glucose, insulin, and NEFAs, and insulin sensitivity as measured by the Cederholm index. We also considered whether the timing of the post-training assessment impacted on the observed metabolic changes. Improvements in glucose, insulin, and NEFA responses were similar whether assessed two or three days following the final training session (mean reduction in glucose AUC: 2 d post 12 ± 10%, 3d post 19 ± 4%; insulin AUC: 2 d post 34 ± 9%, 3d post 38 ± 9%; NEFA AUC, 2d post 23 ± 21%, 3d post 27 ± 9%).

### Intra-individual variation in time trial performance and response to an OGTT

In subjects not performing the HIT program, no significant differences between the first and second OGTTs were observed for plasma glucose (AUC: 671 ± 47 v 659 ± 40 mmol·min·l^-1^) and NEFAs (AUC: 24035 ± 1611 v 22599 ± 2544 μmol·min·l^-1^), nor for time trial performance (1477 ± 214 v 1491 ± 245 s). Coefficients of variation for the repeated measurements were 2.1% for the time trial, 4.9% and 7.0% for fasting glucose and NEFA concentrations, and 8.1% and 8.2% for glucose and NEFA AUCs respectively.

## Discussion

While regular exercise training represents one of the most powerful strategies to reduce the development of metabolic disease in healthy adults [17], most adults fail to meet current guidelines for participation [18]. These guidelines largely focus on the time spent carrying out moderately intense activity (or total energy expenditure), typically require many hours of exercise each week and can fail to modify risk factors relevant for disease prevention [19]. In addition, as time commitment is perceived as a major barrier, driving or contributing to low compliance, then these guidelines may not be the most logical approach for improving public health. In the present study we demonstrate for the first time that only a few minutes of high intensity interval exercise performed over two weeks is required to substantially improve both insulin action and glucose homeostasis in sedentary young males. This is both a physiologically important observation and potentially useful as it highlights a preventative intervention that could logically be implemented as an early strategy to *prevent *age related development of cardiovascular disease.

Interestingly, despite employing long-term training interventions (2–16 months) the majority of studies investigating classic aerobic [10,20-25] or strength training programs [26-28] have observed only a reduction in insulin area under the curve (AUC) in response to a glucose load following training, without a concomitant reduction in glucose AUC, indicating only a partial improvement in insulin action. Furthermore, walking based interventions may not reduce risk factors in the target population where prevention is the key objective [19]. Some longitudinal exercise studies have shown reductions in glucose AUC [29-31], but post-training OGTTs were performed within 24 hours of the last exercise bout and therefore reflect the combined impact of acute and chronic exercise [32]. In contrast, Hughes *et al *demonstrated reduced glucose AUC in elderly subjects without a concomitant change in insulin AUC [33].

The low volume, high intensity training utilized in the current study significantly reduced both glucose AUC (-12%) and insulin AUC (-37%), with a sustained improved insulin action until at least day three after the last exercise session. This was achieved without changes in body weight, and with a weekly energy-'cost' of training of ~225 kcal during the first training week and ~275 kcal during the second training week. This very modest increase in calorie consumption is in stark contrast to the ~2000–3000 kcal·week^-1 ^consumed during a typical aerobic training program [25,34]. This implies, but does not prove, that the mechanism underpinning the benefits we observed with HIT, may be distinct from those responsible for the more modest improvements in insulin action with classic time-consuming aerobic training. While much focus is being given to increasing calorie consumption to ward off weight gain, it is clear that improving metabolic fitness may be just as important as limiting gains body mass index.

Failure for insulin to adequately control blood glucose following a meal is known as 'insulin resistance'. Skeletal muscle is considered the major tissue responsible for the uptake of glucose following a meal, or a glucose or insulin challenge [35] such that it is entirely reasonable to assume that the improvement in glucose and insulin AUC observed in the present study reflected improved muscle glucose uptake. The limiting step in glucose disposal is considered to be its transport into the skeletal muscle [6] and GLUT4 is the most abundant glucose transporter in skeletal muscle. Increased GLUT4 concentration with endurance training has been suggested to be an important factor regulating insulin sensitivity [6,33]. Burgomaster *et al*. reported that skeletal muscle GLUT4 levels increase by ~20% after one week of HIT, and remarkably remained elevated over 6 weeks of training and a subsequent 6-week period of detraining [14]. Given the similarity between our study and the aerobic performance improvement, protocols and subjects in the Burgomaster *et al *studies, these studies should be comparable and thus an increase in GLUT4 may partly explain our findings. However, increased GLUT4 concentration does not always fully explain training-induced improvements in insulin sensitivity and key regulatory proteins down-stream in the insulin signalling pathway are more activated in response to insulin following aerobic training [36]. HIT produces similar changes in skeletal muscle markers of carbohydrate and lipid metabolism to aerobic training [37], so it should be investigated whether HIT also produces similar adaptations of the insulin signalling pathway as seen following traditional aerobic training [36].

Improved whole body glucose disposal following training has been associated with an increase in insulin stimulated glycogen synthesis [38]. HIT has at least two novel features, firstly unlike walking or moderate intensity aerobic training, it involves the activation of a large muscle mass and secondly this is associated with a very high glycogen breakdown-turnover. The combination of these two factors means that a greater proportion of muscle fibres will need to replenish their carbohydrate stores, compared with what would be encountered following moderate intensity aerobic training. Muscle contraction under conditions of metabolic stress (such as incurred during HIT) results in very rapid glycogen degradation [49] and this would almost certainly alter the binding of a variety of glycogen associated proteins [40,41]. Thus we suspect that remodelling of the glycogen pool, altering the molecules' branching architecture [38], may well be important in regulating skeletal muscle insulin sensitivity following HIT. Currently, high intensity muscle contraction is the only feasible strategy for remodelling of the entire muscle glycogen pool in humans.

Insulin sensitivity may also be regulated by plasma NEFA concentration. Pharmacological lowering of plasma NEFA levels has been shown to positively regulate insulin sensitivity during an OGTT [39], whereas raising plasma NEFA concentration, through lipid infusion, lowers glucose infusion rate during peripheral insulinemia-euglycemia in young men [40]. In contrast, exercise training has been shown to have little or no effect on fasting plasma NEFA concentration, insulin mediated lipolysis or NEFA release during exercise [41-44]. In the present study, HIT was associated with a 17% decrease in fasting plasma NEFA concentration without a concomitant change in fasting insulin. Furthermore, there was a 26% reduction in NEFA AUC during OGTT following HIT despite a 37% reduction in the plasma insulin AUC. This suggests that insulin was able to inhibit lipolysis to a greater extent following HIT and while these changes are more modest than those observed with lipid powering drugs, the long terms benefits may still be of significance.

## Conclusion

We demonstrate for the first time that low volume, high intensity interval exercise involving only ~250 kcal of work, is sufficient to achieve significant improvements in glycemic control in sedentary young adults. This study is limited to the measurement of whole body insulin sensitivity which makes the interpretation of our data incomplete. Analysis of tissue specific responses through biopsy studies would enhance the present study by allowing determination of the mechanisms involved in the improvements observed at the whole body level. Nevertheless, our data and the findings by the Gibala lab [4,37,45-49] suggest that current guidelines, with regards to optimizing exercise prescription to yield the best health outcomes, may not be optimal and certainly require further discussion. Our findings warrant further studies investigating the effectiveness of HIT in improving glycemic control in healthy middle aged individuals at risk of developing T2D and in patients with T2D.

## Competing interests

The authors declare that they have no competing interests.

## Authors' contributions

JB and NBJV participated in the study design, analysis, writing and editing of the manuscript, CK, FMG and GC participated in data generation and study analysis. JAT conceived the study idea and participated in the study design, writing and editing of the manuscript.

## Pre-publication history

The pre-publication history for this paper can be accessed here:


